# A STAT-1 knockout mouse model for Machupo virus pathogenesis

**DOI:** 10.1186/1743-422X-8-300

**Published:** 2011-06-14

**Authors:** Steven B Bradfute, Kelly S Stuthman, Amy C Shurtleff, Sina Bavari

**Affiliations:** 1 United States Army Medical Research Institute of Infectious Diseases, Fort Detrick, Maryland, USA

## Abstract

**Background:**

Machupo virus (MACV), a member of the *Arenaviridae*, causes Bolivian hemorrhagic fever, with ~20% lethality in humans. The pathogenesis of MACV infection is poorly understood, and there are no clinically proven treatments for disease. This is due, in part, to a paucity of small animal models for MACV infection in which to discover and explore candidate therapeutics.

**Methods:**

Mice lacking signal transducer and activator of transcription 1 (STAT-1) were infected with MACV. Lethality, viral replication, metabolic changes, hematology, histopathology, and systemic cytokine expression were analyzed throughout the course of infection.

**Results:**

We report here that STAT-1 knockout mice succumbed to MACV infection within 7-8 days, and presented some relevant clinical and histopathological manifestations of disease. Furthermore, the model was used to validate the efficacy of ribavirin in protection against infection.

**Conclusions:**

The STAT-1 knockout mouse model can be a useful small animal model for drug testing and preliminary immunological analysis of lethal MACV infection.

## Background

Machupo virus (MACV), a member of the *Arenaviridae *family, is the causative agent of Bolivian hemorrhagic fever. MACV is spread by inhalation of aerosols generated from excretions of its carrier, the mouse *Calomys callosus*, although human-to-human transmission can also occur [1-3]. Case fatality rates are approximately 20% in humans. The disease course in humans is similar to other New World arenavirus infections, such as Junín virus infection, with an incubation period of ~1-2 weeks, followed by fever and malaise, headache, dizziness, back pain, petechia, erythema, and myalgia (reviewed in [1]). Leukopenia, thrombocytopenia, hemorrhaging, and neurological symptoms are often prominent. Patients that succumb to infection generally do so 7-12 days after onset of symptoms. Subcutaneous inoculation of non-human primates (NHPs) results in a fairly similar disease course to that seen in humans, with death ranging from 8-25 days after virus exposure [4-9]. NHPs that survive past this time point develop neurological symptoms, with some succumbing to disease and others surviving. Histopathologic comparison of tissue from NHPs or humans that succumb to MACV infection revealed hemorrhaging and necrosis in various organs (with some differences between human and NHP findings), but none of these manifestations were thought to be severe enough to cause death [6,10].

Early studies showed that MACV was also lethal in adult guinea pigs, suckling mice and hamsters [5]. However, there are only minimal reports describing MACV pathogenesis in animals. In NHP models, while limited viremia data are available, only minimal work has been done to describe MACV pathogenesis, and virtually no studies have analyzed immune responses to infection. Guinea pigs can be lethally infected with MACV, but features of the disease course in these animals are almost completely uncharacterized, as data on viral replication, pathology, and host response are lacking [5]. MACV is not lethal in adult immunocompetent mice [5], making characterization of the disease and evaluation of candidate therapeutic compounds difficult. Recently, mice lacking interferon responses have been used as models for infection with other hemorrhagic fever viruses, including Ebola, Marburg, Junín, and Crimean-Congo Hemorrhagic Fever viruses [11-14]. Signal transducer and activator of transcription 1 (STAT-1) knockout mice are defective in type I, II, and III interferon signaling. The objective of this study was to test STAT-1 knockout mice as an adult mouse model for MACV pathogenesis and immunity.

## Methods

### Mice and infection

Male and female STAT-1 knockout mice, 6-12 weeks old, were obtained from Taconic Farms. Infections were performed with approximately 1000 PFU of wild-type Machupo virus (strain Carvallo) which had been passaged 3-4 times in suckling hamster brain and subsequently twice in Vero cell culture. For lethality studies, mice were scored at least twice daily for health and appearance, and any moribund mice were euthanized. For serial sampling studies, four mice were euthanized before (day 0) and on days 3, 5, and 7 after virus challenge. This was done twice, and the data were pooled. Research performed at The United States Army Medical Research Institute of Infectious Diseases (USAMRIID) was conducted under an IACUC approved animal protocol in compliance with the Animal Welfare Act and other federal statutes and regulations relating to animals and experiments involving animals and adheres to principles stated in the *Guide for the Care and Use of Laboratory Animals *(National Research Council, 1996). USAMRIID is fully accredited by the Association for the Assessment and Accreditation of Laboratory Animal Care International. All virus work was performed and all infected mice were handled under maximum containment in a biosafety level-4 laboratory at USAMRIID.

### Hematology and Chemistry analysis

Blood samples from anesthetized mice were collected, by cardiac puncture, in EDTA tubes. For hematology analysis, blood was analyzed with a Beckman Coulter ACT 10 counter. Whole blood was further processed by centrifugation at 10,000 RPM for 1 minute for plasma isolation, which was then tested using comprehensive metabolic panels and an Abaxis Piccolo chemistry analyzer.

### Histopathologic sampling

Four animals were randomly chosen for gross necropsy on days 0, 3, 5, and 7. Tissues were collected in 10% neutral buffered formalin and held in the biosafety-level-4 laboratory for > 21 days. Tissues were then embedded in paraffin, thin sectioned for histology, and stained with hematoxylin and eosin for routine light microscopy.

### Ribavirin studies

Groups of 5 STAT-1 knockout mice were administered 100 mg/kg ribavirin (in water) intraperitoneally 1 hour after infection with 1000 pfu MACV. A control group received water. Each group received daily injections through day 12 and was monitored for survival. This study was performed twice and data were pooled.

### Statistical analysis

Two-tailed T-tests were performed to compare test groups to day 0 samples, and statistical significance is denoted where p ≤ 0.05.

### Cytokine analysis

Plasma cytokine levels were measured using a Cytokine Bead Array Flex Set kit (BD Biosciences) according to the manufacturer's protocol. Samples were analyzed on a BD FACSCanto II.

## Results

**Figure 1:**
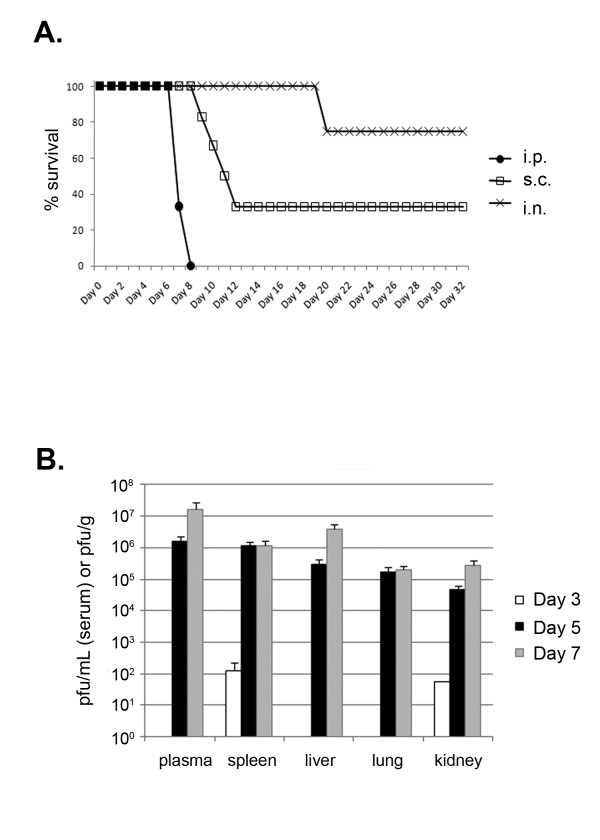
**Wild-type MACV is lethal in STAT-1 knockout mice**. A) STAT-1 knockout mice were infected with 1000 pfu MACV via the intraperitoneal (n = 6), subcutaneous (n = 6), or intranasal route (n = 4) and monitored for survival. Intraperitoneal infection was used for subsequent studies. B) Tissue samples were harvested at days 3, 5, and 7 after MACV infection and evaluated for viral load (plasma, n = 7-8; spleen, liver, lung, kidney n = 4).

The disease progression and lethality of MACV infection was assessed in STAT-1 knockout mice. Infection of STAT-1 knockout mice with ~1,000 pfu of MACV via the intraperitoneal route resulted in lethality (defined by either death, or euthanasia of moribund mice) in 6/6 mice (mean time to death (MTD) 7.3 ± 0.5) (Figure 1A). Mice began to appear ruffled, hunched, and lethargic approximately five days after infection. Subcutaneous infection of STAT-1 knockout mice resulted in death in 4 of 6 mice, with a delayed time-to-death (MTD 10.5 ± 1.3), while intranasal infection was lethal in only 1 of 4 mice (death on day 20). Therefore, intraperitoneal infection appeared to be the most uniformly lethal route of administration, and was performed in subsequent sampling and drug evaluation studies.

Serial sampling studies (days 0, 3, 5, and 7 post-infection) were performed to investigate the pathogenesis of MACV lethal infection in STAT-1 knockout mice. Viral titers were detected in spleen and kidney on day 3 at low levels (≈100 pfu/g tissue) (Figure 1B). By days 5 and 7, virus had spread to other organs, and higher viral titers of between 10^5 ^and 10^7 ^pfu/g tissue (or pfu/mL plasma) were detected in all tissues analyzed (plasma, spleen, lung, liver, and kidney).

**Figure 2:**
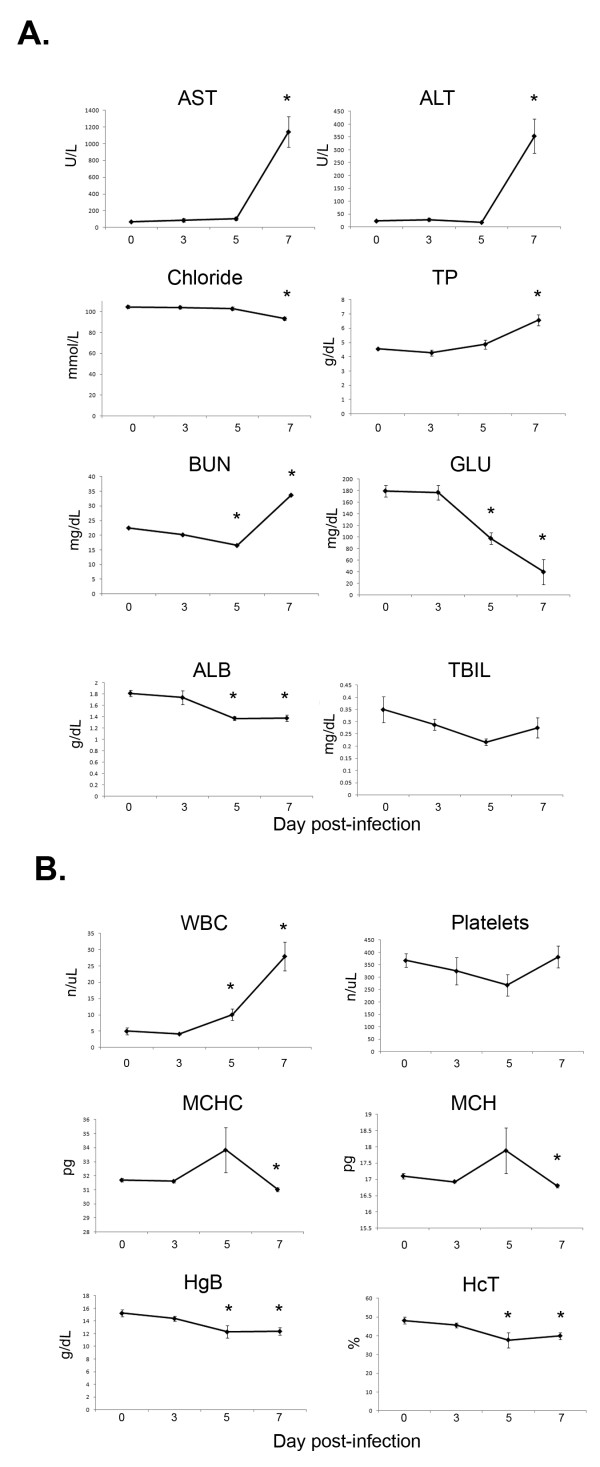
**Hematology and plasma chemistry analysis**. A) Plasma was harvested on days 0, 3, 5, and 7 after MACV infection and analyzed for changes in plasma chemistry parameters. B) Whole blood was analyzed for white blood cell and platelet counts, as well as hemoglobin and hematocrit levels. *p < 0.05.

Plasma was analyzed for evidence of metabolic alterations during infection. Concentrations of both alanine aminotransferase (ALT) (which is often indicative of damage to hepatocytes) and aspartate aminotransferase (AST) (which is indicative of damage to cells of major organs such as the liver, heart, skeletal muscle, kidney, and brain) rose dramatically on day 7 (Figure 2A). Additionally, total protein concentration increased, which can be associated with inflammation. Albumin concentration dropped significantly, which can be indicative of inflammation and shock. Blood urea nitrogen also rose on day 7 after falling on day 5. Chloride concentration dropped slightly on day 7, while glucose dropped on days 5 and 7 relative to day 0 levels (Figure 2A). The pattern of changes for these three parameters points to possible liver and kidney damage and malfunction, although further research should be conducted to confirm this hypothesis. No significant changes were observed in total bilirubin concentration.

Hematological analysis revealed a substantial increase in white blood cell counts during the course of infection (Figure 2B). Platelet levels decreased somewhat on day 5 post-infection (p = 0.073) before rebounding on day 7. Interestingly, hemoglobin and hematocrit levels slightly decreased as infection progressed, as did mean corpuscular hemoglobin (MCH) and mean corpuscular hemoglobin concentration (MCHC) levels (Figure 2B).

**Figure 3:**
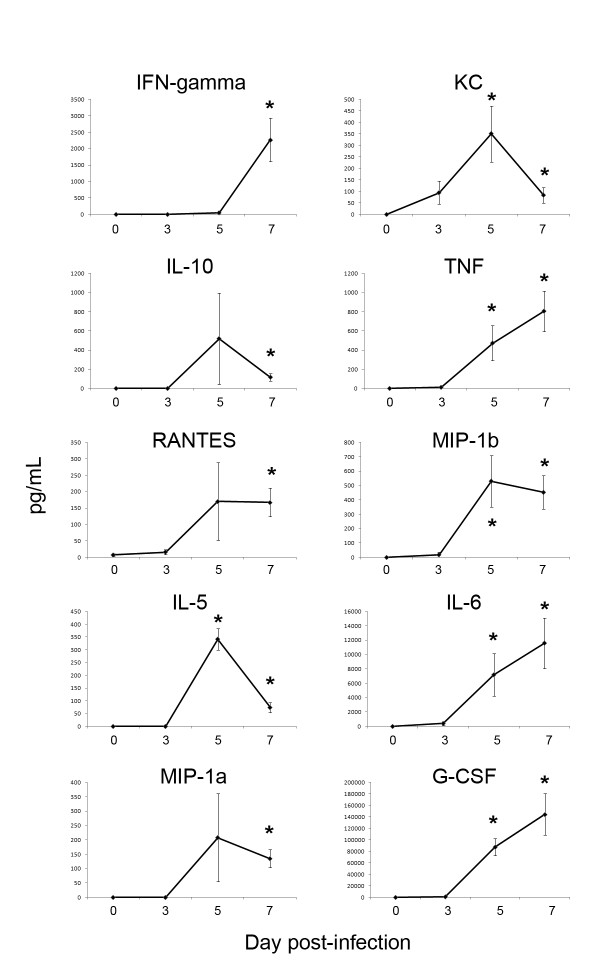
**Cytokine and chemokine analysis**. Concentrations of 17 different cytokines and chemokines in plasma was evaluated on days 0, 3, 5, and 7 post-infection (n = 6-7). *p < 0.05.

Cytokine and chemokine levels were analyzed in plasma from MACV-infected STAT-1 knockout mice (Figure 3). The levels of IFN-gamma, IL-5, KC (mouse orthologue of IL-8), IL-6, IL-10, MIP-1 alpha, MIP-1 beta, TNF-alpha, G-CSF, and RANTES were all elevated on days 5 and/or 7, relative to day 0. However, no changes were detected in IL-3, IL-4, IL-9, IL-21, GM-CSF, IL-17A, or IL-1 beta (data not shown).

**Figure 4:**
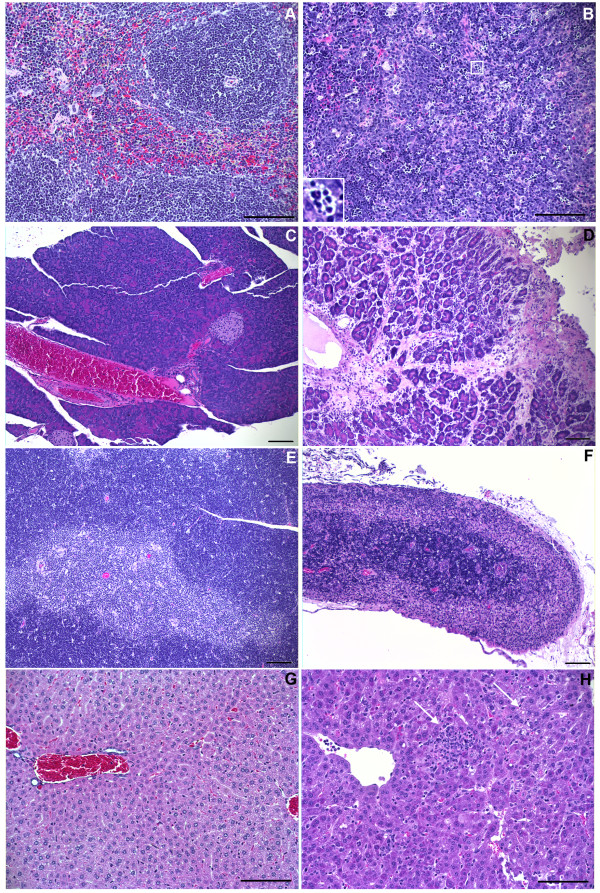
**Pathology**. Panels on the left of the figure (A, C, E and G) show representative photomicrographs of H&E stained sections of spleen, pancreas, thymus, and liver, respectively, removed from uninfected mice at day 0. Panel B shows the representative disruption of the splenic white pulp architecture at day 7 post-infection, with marked lymphocyte death (example in enlarged inset). Panel D demonstrates pancreatitis, which was observed in all 4 animals at day 7 post infection. Panel F is an example of the complete thymic cortical atrophy observed in all day 7 post-infection mice. Panel H is a representative sample of day 7 liver, demonstrating mild to moderate areas of inflammation and necrosis (left arrow) and regions of hepatocellular degeneration and cell death (right arrow) (Panels A, B, G and H at 200x magnification, scale bar is 50 microns; Panels C, D, E and F, scale bar is 10 microns).

Tissues were taken from MACV-infected STAT-1 knockout mice and analyzed for histopathological changes (Figure 4). Mild to moderate hepatocellular degeneration and necrosis were found in day 7 animals, consistent with liver injury (Figure 2A). Lymphocyte death was present in thymus, spleen, and lymph nodes as early as day 3, and increased as infection progressed through day 7. Mice presented extensive thymic cortical atrophy, and moderate to marked splenic lymphocyte death, by day 7. Another prominent histological finding was variably extensive peritonitis and necrotizing steatitis found in day 5 and day 7 animals. The lesions were most prominent in and around pancreatic lobes but the inflammation did not appear to involve the pancreas in day 5 animals; however all animals from day 7 termination had mild to marked pancreatitis.

**Figure 5:**
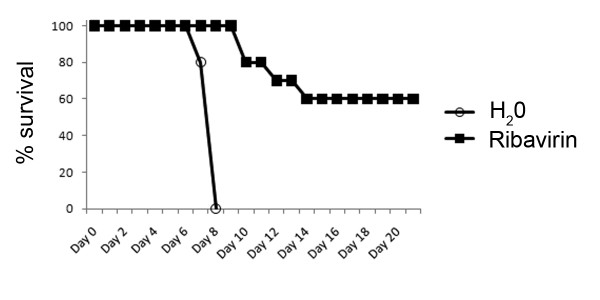
**Efficacy of ribavirin against MACV infection**. STAT-1 knockout mice were infected with MACV and treated daily with ribavirin or vehicle (water) beginning 1 hour after infection (n = 10).

We also investigated the suitability of the MACV STAT-1 knockout model for testing antiviral therapeutics. Ribavirin has been previously used to treat two human patients infected with MACV [15]; although both survived, it has not been conclusively shown that ribavirin was responsible for protection. A recent study demonstrated partial efficacy of ribavirin in guinea pigs infected with MACV [16]. To test the efficacy of ribavirin, and to demonstrate the utility of this model for drug discovery studies, STAT-1 knockout mice were infected with MACV, and ribavirin (100 mg/kg/day) or water was administered i.p. beginning one hour post-infection and continuing daily for 12 days. As shown in Figure 5, ribavirin treatment protected 60% of mice from death, and the mice that succumbed to infection had a significantly later mean time-to-death than untreated mice (11.5 ± 1.0 days for ribavirin treated vs. 7.8 ± 0.1 days for water treated mice; p < 0.01). These antiviral efficacy data support the use of STAT-1 knockout mice as a platform to test antiviral therapeutics for efficacy against MACV infection.

## Discussion

In this study, STAT-1 knockout mice were infected with MACV, resulting in a lethal small animal model for this arenavirus. To date, lethal models of MACV infection which seem to somewhat accurately model human infections have been limited to NHPs [5,6,9,17,18]. This underscores a need for a small animal model to enable collection of data for a better understanding of MACV immunopathogenesis, and as a tool for antiviral therapeutics discovery. Because arenaviruses can block host IFN responses to establish infection, mice lacking elements of the interferon response pathway were attractive candidates for development of small animal models. It has recently been demonstrated that the Z protein of New World arenaviruses (including MACV) binds to the retinoic acid-inducible gene I (RIG-I) protein and subsequently inhibits IFN-beta responses [19]. Additionally, MACV nucleoprotein blocks nuclear translocation of interferon regulatory factor 3 (IRF-3) and inhibits activation of promoters dependent on IRF-3 and IFN-beta [20]. Therefore, our data are in agreement with these studies to show that type I IFN responses may be crucial for control of MACV infection.

The intraperitoneal route of infection of STAT-1 knockout mice with MACV resulted in a much more rapid and lethal disease course compared to subcutaneous or intranasal routes (Figure 1A). Published NHP studies for MACV infection have been conducted with subcutaneous infection, which results in death ranging from ~8-30 days [5-7,21]. Interestingly, subcutaneous infection of NHPs does not consistently result in uniform lethality, which is in agreement with what is reported here. Future studies could investigate whether an alternate route of infection of NHPs would alter MACV pathogenesis, leading to a change in time to death.

Virus was detected in the plasma and organs of MACV infected STAT-1 mice. The virus appeared to have an early tropism for the spleen and kidney (day 3) in this rodent model (Figure 1B). MACV spread by day 5 to all other organs sampled, and titers remained high until the time of death (day 7). To our knowledge, this is the first study to analyze MACV viral titers in tissues at different points after infection.

Very little data have been published for clinical chemistry values in plasma of MACV-infected animals or patients. One study reported various parameters at different time points for only 3 patients, although baseline values for these individuals were not available [15]. This study suggested elevated AST and ALT values after presentation of symptoms; BUN and creatinine levels were moderately elevated or normal. While hamsters, marmosets, African green monkeys and rhesus monkeys have all been described to be susceptible to MACV infection, very few clinical chemistry values were reported and therefore comparative analysis is challenging. There was one report of normal bilirubin levels in rhesus monkeys, which the authors believed to reflect little to no intravascular hemorrhage in this model at that time point [9]. The data reported here comprise the first study to present a dataset from an animal model for several important clinical chemistry parameters. There is evidence of liver and possible kidney damage in MACV infected STAT-1 mice, as revealed by significantly elevated AST, ALT, BUN, and decreased chloride, glucose, and albumin levels (Figure 2A). Total bilirubin did not change in these animals, similar to what was noted in rhesus monkeys [9]. A more thorough assessment of values for clinical chemistry parameters across various species of animal models, as well as human infections, is needed.

Total white blood cell counts increased over the course of MACV infection of STAT-1 knockout mice (Figure 2B). In NHP or human MACV infections, however, white blood cell count (or lymphocyte and neutrophil counts) decline before recovering [9,17]. Additionally, thrombocytopenia is a hallmark of human and NHP MACV infection [2,9,22], but only mild decreases in platelet counts were found in STAT-1 knockout mice (p = 0.073 on day 5; Figure 2B). Following MACV infection, hematocrit levels declined in STAT-1 knockout mice (Figure 2B). Importantly, decreased hematocrit levels have been reported in rhesus and African green monkeys [9,17]. STAT-1 knockout mice also had decreased MCH, MCHC, and hemoglobin levels during infection, which can be indicative of the observed decreased hematocrit levels.

Virtually no work has been reported on cytokine production in response to MACV infection. The data reported herein suggest a dysregulated cytokine/chemokine response towards the end of lethal MACV infection, with elevated pro- and anti-inflammatory mediators (Figure 3). It is noteworthy that elevated levels of IL-6, IL-8, IL-10, G-CSF, and TNF-alpha, which were observed in MACV-infected STAT-1 knockout mice (Figure 3), are correlated with the severity of human Argentinean hemorrhagic fever, caused by the related Junín virus [23-25]. It has been hypothesized that one mechanism of hemorrhagic fever virus pathogenesis is late stage elaboration of uncontrolled pro-inflammatory cytokine responses and release of vasoactive mediators. These mediators may contribute to decreases in endothelial cell function leading to vascular leakiness, although this mechanism has yet to be precisely described for arenaviruses [26]. Although it is probable that cytokine levels in STAT-1 knockout mice will be altered due to their inability to respond to type I, II, and III IFNs, the cytokine data reported here can serve as a reference for future MACV studies in other immunocompetent animal models.

We completed a histopathological examination of all thoracic and abdominal organs, as well as brain and cephalic tissues. The most significant histopathological findings were mild to moderate liver damage, moderate to marked lymphocyte death in lymph nodes, spleen and thymus, and pancreatitis (Figure 4). These findings have also been reported to varying degrees in other MACV models. Lymphoid depletion in the splenic white pulp was described for MACV-infected rhesus monkeys [6], while infected marmosets demonstrated cortical necrosis of lymph nodes and splenic reticular hyperplasia with lymphoid depletion [5]. MACV-infected African green monkeys showed moderate hepatocellular necrosis with fatty changes, mild to moderate pancreatitis, and mild to moderate lymphoid cell depletion with necrosis in the spleen, lymph nodes and thymus [7]. There have not been additional comparable histopathological analyses performed in MACV NHP models in decades.

NHPs tend to have a biphasic disease course after MACV infection, with most deaths occurring in the first "acute" phase. Surviving NHPs tend to undergo a second, prolonged disease state, characterized by neurological disease [21]. This second phase has not been described in human MACV infection (to our knowledge) or in the STAT-1 knockout model. However, due to a lack of data, it is not feasible to conclusively compare the faithfulness of different MACV infection models to each other or the human disease course. Overall, there are important similarities and differences in the disease course of these models, and further work must be done to directly compare changes in STAT-1 knockout mice to NHP models or human infections.

The STAT-1 knockout mouse model was evaluated by testing the efficacy of ribavirin against MACV disease. The antiviral drug ribavirin has been shown to be protective in MACV-infected guinea pigs [16], and has been used to treat two human MACV-infected patients [15]. The results reported here indicate that ribavirin protected STAT-1 knockout mice against MACV disease, verifying the efficacy of ribavirin and supporting the utility of the STAT-1 knockout model for drug efficacy studies (Figure 5).

Mice lacking type I and II IFN signaling (IFNalpha/beta receptor KO/IFN-gamma receptor KO mice) have been shown to be susceptible to Junín virus infection, leading to weight loss of >15-20%, at which point mice were euthanized [13]. Splenic necrosis and lymphocyte depletion were not apparent in these mice, but kidney and liver inflammation and necrosis were prominent. MACV-infected STAT1 KO mice also had liver necrosis, but in contrast to the Junín virus model, splenic lymphocyte death was prevalent. Further work is necessary to compare how similar these mouse models are to each other and to their respective human disease courses.

## Conclusions

These data suggest the STAT-1 knockout mouse is a useful small animal model for investigation of efficacy of antiviral therapeutics as well as preliminary studies of immune responses to MACV infection. MACV infections in mouse, guinea pig and NHP species should be characterized in more detail to provide well-understood models for investigation of therapeutics and vaccines. Further characterization of these models is required to advance the development of countermeasures for arenaviral hemorrhagic fevers.

## Competing interests

The authors declare that they have no competing interests.

## Authors' contributions

SBB designed the study, drafted the manuscript, and performed experiments. KS performed experiments. AS performed experiments and drafted the manuscript. SB provided supervision on experimental design and manuscript preparation. All authors read and approved the final manuscript.
